# Can the microRNA expression profile help to identify novel targets for zoledronic acid in breast cancer?

**DOI:** 10.18632/oncotarget.8722

**Published:** 2016-04-13

**Authors:** Daniele Fanale, Valeria Amodeo, Viviana Bazan, Lavinia Insalaco, Lorena Incorvaia, Nadia Barraco, Marta Castiglia, Sergio Rizzo, Daniele Santini, Antonio Giordano, Sergio Castorina, Antonio Russo

**Affiliations:** ^1^ Department of Surgical, Oncological and Oral Sciences, Section of Medical Oncology, University of Palermo, Palermo, Italy; ^2^ University Campus Bio-Medico, Department of Medical Oncology, Rome, Italy; ^3^ Sbarro Institute for Cancer Research and Molecular Medicine, Center for Biotechnology, College of Science and Technology, Temple University, Philadelphia, PA, USA; ^4^ Fondazione Mediterranea “G.B. Morgagni”, Catania, Italy; ^5^ Department of Biomedical and Biotechnological Sciences, University of Catania, Catania, Italy

**Keywords:** bone metastasis, breast cancer, microarray analysis, miRNA expression profile, zoledronic acid

## Abstract

Zoledronic acid (ZOL), belonging to third generation bisphosphonate family, is a potent inhibitor of osteoclast-mediated bone resorption, widely used to effectively prevent osteolysis in breast cancer patients who develop bone metastases. Low doses of ZOL have been shown to exhibit a direct anticancer role, by inhibiting cell adhesion, invasion, cytoskeleton remodelling and proliferation in MCF-7 breast cancer cells. In order to identify the molecular mechanisms and signaling pathways underlying the anticancer activity exerted by ZOL, we analyzed for the first time the microRNA expression profile in breast cancer cells. A large-scale microarray analysis of 377 miRNAs was performed on MCF7 cells treated with 10 μM ZOL for 24 h compared to untreated cells. Furthermore, the expression of specific ZOL-induced miRNAs was analyzed in MCF-7 and SkBr3 cells through Real-time PCR. Low-dose treatment with ZOL significantly altered expression of 54 miRNAs. Nine upregulated and twelve downregulated miRNAs have been identified after 24 h of treatment. Also, ZOL induced expression of 11 specific miRNAs and silenced expression of 22 miRNAs. MiRNA data analysis revealed the involvement of differentially expressed miRNAs in PI3K/Akt, MAPK, Wnt, TGF-β, Jak-STAT and mTOR signaling pathways, and regulation of actin cytoskeleton. Our results have been shown to be perfectly coherent with the recent findings reported in literature concerning changes in expression of some miRNAs involved in bone metastasis formation, progression, therapy resistance in breast cancer. In conclusion, this data supports the hypothesis that ZOL-induced modification of the miRNA expression profile contributes to the anticancer efficacy of this agent.

## INTRODUCTION

Breast cancer (BC) is the most common cancer and the major cause of cancer death in women worldwide [1, 2]. The mortality associated to BC is correlated with bone metastasis in about 50% of patients, therefore, maintaining bone integrity is important for these patients [3]. Metastatic bone disease (MBD) is a painful complication characterized by elevated rates of localized osteolysis, which can lead to potentially debilitating skeletal-related events (SREs) such as pain, pathological fracture, hypercalcaemia and spinal cord compression [4, 5]. Interference in the complex interactions between tumor and bone cells in the bone microenvironment results in MBD. Cytokines and growth factors produced by tumor cells destroy the balanced process between osteoclastic bone resorption and osteoblastic bone formation causing increased osteolysis. The alteration and disruption of the normal surrounding extracellular matrix (ECM), induced by matrix metalloproteinases (MMPs), determines the primary tumor cell invasion leading to development of bone metastasis [6, 7]. This event causes the extravasation of cancer cells reaching distant target organs including skeletal tissue, where they establish respective metastases [8].

**Figure 1:**
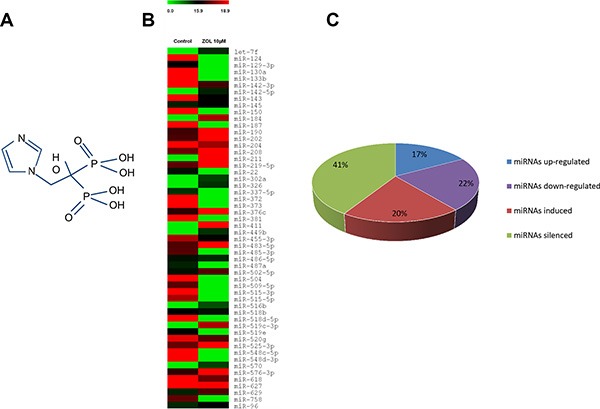
MiRNA expression profile induced by ZOL (**A**) Chemical structure of zoledronic acid. (**B**) Heat map of differentially expressed miRNAs by ZOL in breast cancer cells. The heat map was generated from microarray data reflecting expression values in MCF-7 cells treated with 10 μM ZOL for 24 h in comparison to untreated cells (control). Only up-regulated miRNAs with fold change > 2 and down-regulated miRNAs with fold change < 0.3 were considered (*P* < 0.05). Each row represents the expression levels for a single miRNA tested for two different experimental conditions. Each column shows the expression levels for the miRNAs tested for a single experimental condition. The absolute expression value of each miRNA is derived from the mean of two biological replicates. The color scale bar on the top represents signal intensity variations ranging from green (poorly expressed or unexpressed miRNAs) to red (highly expressed miRNAs). Black boxes indicate intermediate expression values. (**C**) Pie chart representation of the 54 differentially expressed miRNAs obtained by miRNAs expression profile: 9 up-regulated miRNAs, 12 down-regulated miRNAs, 11 miRNAs induced by ZOL and 22 silenced miRNAs in MCF7 treated with ZOL.

Bisphosphonates (BP) are potent inhibitors of osteoclast-mediated bone resorption approved for the treatment of MBD from advanced cancers, including BC [9]. Zoledronic acid (ZOL) is a third generation nitrogen-containing BP (N-BP) (Figure 1A) used for treatment of BC patients with osteolytic lesions in order to significantly reduce the risk of skeletal complications. N-BPs inhibit farnesyl bisphosphate (FPP) synthase, whose activity is crucial in the mevalonate pathway, by preventing lipid prenylation of small GTPases, such as Ras, Rho and Rac, and, consequently, blocking downstream signaling pathways and inducing apoptosis of osteoclasts and tumor cells [10–13]. Recent data suggested that ZOL, alone or in combination with neo-adjuvant chemotherapy, exerts direct or indirect anticancer effects on a variety of cancers, including BC [14–16]. In particular, the *in vitro* anticancer activity of ZOL is correlated to reduced migration, invasion, adhesion and proliferation and increased apoptosis of cancer cells [17–20]. ZOL also exhibits indirect anticancer activities, by inhibiting angiogenesis [21] and tumor-associated macrophage infiltration and promoting cytotoxicity of γδ T cells [22–24].

Using a microarray platform, we previously demonstrated that ZOL could modulate the expression of genes involved in metabolic processes, cytoskeletal and ECM organization, cell communication and cell proliferation pathways in MCF7 BC cell line. Therefore, ZOL has been shown to inhibit the invasiveness processes in cancer cells by modifying their capability to invade tumor microenvironment and thus turning off their metastatic potential [25]. We found also that low doses of ZOL block cellular proliferation, by inhibiting the phosphorylated state of AKT and MAPK proteins, and affect the cytoskeletal reorganization by up-regulating fibronectin-1 (FN1) and actin. Moreover, we observed that ZOL treatment promotes the TGF-β1/SMADs pathway and mediates the anti-angiogenic potential in MCF7 cells via up-regulation of the thrombospondin-1 (THBS1) expression [26].

MicroRNAs (miRNAs) are a group of non-coding regulatory small RNAs, 20–22 nucleotides in length, which have been shown to regulate several cellular processes such as proliferation, differentiation, apoptosis, cell metabolism and angiogenesis [27]. MiRNAs can inhibit gene expression by recognizing specific binding sites in the 3ʹuntranslated region (UTR) of target mRNA molecules [28], leading to their degradation, inhibition of their translation, or both [27, 29]. The dysregulated expression of miRNAs, observed in almost all human malignancies, is involved in several cancer processes, including cell cycle control, angiogenesis, metastasis, apoptosis, invasion, and resistance to hypoxia [30–33]. Different miRNA expression profiles were associated with specific BC pathologic characteristics, such as tumor stage, progesterone and estrogen receptor expression, vascular invasion and proliferation index [34]. Recent studies showed that several miRNAs are involved in bone metastases formation, by interfering with the crucial steps of cancer cell intravasation and tumor invasion, and targeting specific genes implicated in epithelial-mesenchymal transition (EMT), survival, invasiveness, motility, osteomimicry and bone remodeling [35, 36]. Many miRNAs were identified as mediators of bone metastases acting as oncomiR (miR-17-92, miR-373, miR-520c) or anti-oncomiR (miR-7, miR-30, miR-34a, miR-143, miR-145, miR-335) in tumor invasion processes [37]. In a recent paper, Croset et al. [38] have grouped the main miRNAs involved in bone metastasis development into three following processes: bone remodeling (miR-33a and miR-326), osteomimicry (miR-30s family, miR-204, miR-211, miR-218 and miR-379) and EMT (Let-7 family, miR-7, miR-10b, miR-34a, miR-100, miR-143, miR-145, miR-200 family, miR-203 and miR-205). Specific miRNAs involved in regulation of BC bone metastases were found to be up-regulated (miR-10b, miR-21, miR-135a, miR-155, miR-221/222, miR-224, miR-373 and miR-520c) and down-regulated (miR-30s, miR-31, miR-34a, miR-125, miR-200, miR-203, miR-205, miR-206 and miR-342). Five miRNAs of the miRNA-30s family are specifically involved in BC cell dissemination to bone, by modulating expression of osteomimetic genes such as *Runx2*, *connexin 43*, *integrin-β3*, *cadherin-11* and *CTGF* [39].

The data on miRNA expression profiles in human cancer demonstrates that miRNAs are promising predictive, prognostic and/or diagnostic markers [40, 41]. Specific cancer-related miRNA expression patterns could be very helpful to evaluate the efficacy of several drugs in the treatment or prevention of tumors and allow to elucidate underlying molecular mechanisms.

Considering the antitumoral activity of ZOL in BC and the important role of miRNAs in regulating different cellular networks, we analyzed the miRNA expression profile in MCF7 BC cell line after treatment with low doses of ZOL to better understand if and how the molecular mechanism by which ZOL mediates these effects involves miRNAs. Only one dose of ZOL was used, because in our previous work [26] three different concentrations (10, 50 and 100 μM) at three different time-points (24, 48 and 72 h) were tested, and the lower dose (10 μM) and the best time-point (24 h), sufficient to induce an anti-proliferative effect on MCF-7 cells, were identified by *in vitro* cell viability assays (data not shown). We performed an expression study on a set of 377 human miRNAs and validated the expression of 11 specific ZOL-induced miRNAs in MCF-7 and SkBr3 BC cells. For the first time, we showed a panel of differentially expressed miRNAs in cells treated with ZOL compared to untreated cells and established, at the light of previous our results [26], the correlation between these miRNAs and cancer-related pathways.

## RESULTS

### MiRNA expression profile induced by ZOL

Understanding mechanisms underlying miRNAs function in the cancer onset and progression provides an useful tool to develop different strategies able to use miRNAs as potential targets for cancer treatment. Since several experimental studies reported that miRNAs may be significant diagnostic and prognostic biomarkers in human tumors, alterations in miRNA expression patterns of different cancers can be associated to specific pathological aspects, disease outcome and treatment response. Nowadays, there is no evidence of dysregulated miRNAs in response to ZOL treatment, therefore it would be original and intriguing to identify the molecular mechanisms allowing their use in anticancer therapeutic strategies. In order to understand the effect of low-dose ZOL treatment on miRNA expression profile we performed a large-scale analysis of 377 miRNAs on MCF7 cells treated with 10 μM ZOL for 24 h compared to untreated cells.

Among 377 human miRNAs, about 40% of them (150) was not detected in MCF-7 cells. Analysis showed 205 microRNAs expressed in MCF7 BC cells after treatment with 10 μM ZOL for 24 h (data not shown). This result suggests that only a part of the miRNA population is expressed in human MCF-7 BC cells. Accumulating evidence showed that several miRNAs are expressed in a tissue- or species specific manner and only a small amount of miRNAs is expressed in a specific tissue at a determined time [42]. Our result is consistent with this conclusion.

In order to highlight the significantly expressed miRNAs we established a cut off of fold change > 2 for up-regulated miRNAs and < 0.3 for down-regulated miRNAs. Statistical analysis revealed 54 miRNAs differentially expressed in MCF7 cells treated with 10 μM ZOL compared to control cells (Figure 1B). Among these 54 miRNAs, we identified 9 up-regulated miRNAs, 12 down-regulated miRNAs, 11 miRNAs specifically induced by ZOL treatment and 22 silenced miRNAs in MCF7 cells treated with ZOL (Figure 1C).

### Functional analysis of miRNAs up- and down-regulated by ZOL treatment

In order to investigate the biological role of differentially expressed miRNAs in MCF7 cells treated with 10 μM ZOL, we used mirPath software. Nine up-regulated and twelve down-regulated miRNAs with different expression fold changes in comparison to untreated cells were found (Figure S1). For statistical analysis only the intersection of targeted genes (hypothetical genes targeted by all selected miRNAs) was evaluated [43].

The obtained results showed the involvement of ZOL in PI3K-Akt signaling pathway, one of the most significant pathways in cancer biology. Statistical analysis showed that 6 ZOL-deregulated miRNAs, such as miR-142-3p, miR-483-5p, miR-486-5p, miR-502-5p, miR-627 and miR-96-5p, shared 60 genes involved in the PI3K-Akt signaling pathway (Table 1). The phosphorylation process of AKT and ERK1/2 can be inhibited by up to 75% and 36%, respectively, after 24 h of ZOL treatment [44]. The extent of inhibition of phosphorylated-protein kinase B (p-AKT) and phosphorylated-mammalian target of rapamycin (p-mTOR) was responsible for inhibitory effect of ZOL on cell growth [45]. Moreover, we showed that also low-dose ZOL treatment (10 μM) reduces both MAPK and Akt activities, by which ZOL slows the cell proliferation and spread of cancer cells after they have colonized bone [26].

**Table 1:** Hypothetical gene targets of 6 miRNAs deregulated by ZOL in MCF7

PI3K/Akt signaling pathway			
PRLR	BCL2	PIK3R1	PIK3CA
PDGFRA	CDKN1B	JAK3	CREB3L1
IFNA4	PPP2R5D	PIK3CG	FOXO3
ITGB8	GNB1	FGF9	IFNA7
NRAS	COL6A6	IRS1	FN1
PPP2R3A	KRAS	RAC1	IFNA17
YWHAE	CDK6	IFNA16	FGF23
THBS2	IL7R	FGF18	MTOR
PIK3R2	GHR	LAMC1	ITGA6
PIK3R5	IKBKB	IGF1	TNN
YWHAG	BRCA1	BCL2L1	PTEN
CREB1	ITGAV	PDGFC	MAPK1
GNG12	GNB2	CREB3L2	IFNA10
GNB3	JAK2	IFNA14	FGF7
PIK3AP1	EIF4E	PDGFD	GRB2

The table represents 60 supposed targets of 6 differentially expressed miRNAs in MCF7 cells after low-dose ZOL treatment. The list was generated by DIANA-miRPath v2.0. *p* < 0.005.

We identified other pathways such as lysine degradation (10 genes, 4 miRNAs), Wnt signaling (22 genes, 5 miRNAs), TGF-β signaling (11 genes, 4 miRNAs), Jak-STAT signaling (24 genes, 5 miRNAs), regulation of actin cytoskeleton (36 genes, 3 miRNAs) and mTOR signaling (19 genes, 3 miRNAs) (Table 2).

**Table 2:** Cellular pathways modulated by 21 differentially expressed miRNAs in MCF7 cells treated with ZOL

Pathway	miRNAs	Genes
PI3K /Akt signaling pathway	6	60
Lysine degradation	4	10
Wnt signaling pathway	5	22
TGF-β signaling pathway	4	11
Jak-STAT signaling pathway	5	24
Regulation of actin cytoskeleton	3	36
mTOR signaling pathway	3	19

The pathways were obtained using DIANA-miRPath v2.0. The first column describes the pathway, the middle column reports the number of miRNAs involved in the same pathway and the last column the number of target genes. *p* < 0.005.

Actin reorganization [26], cell cycle progression [46], apoptosis [47], angiogenesis [48], DNA repair [49, 50], NF-kB signaling [51] are elucidated pathways through which ZOL exerts its anticancer activity. Our analysis showed that miRNAs are possible molecular mediators of these effects.

### ZOL induces expression of specific miRNAs

**Figure 2:**
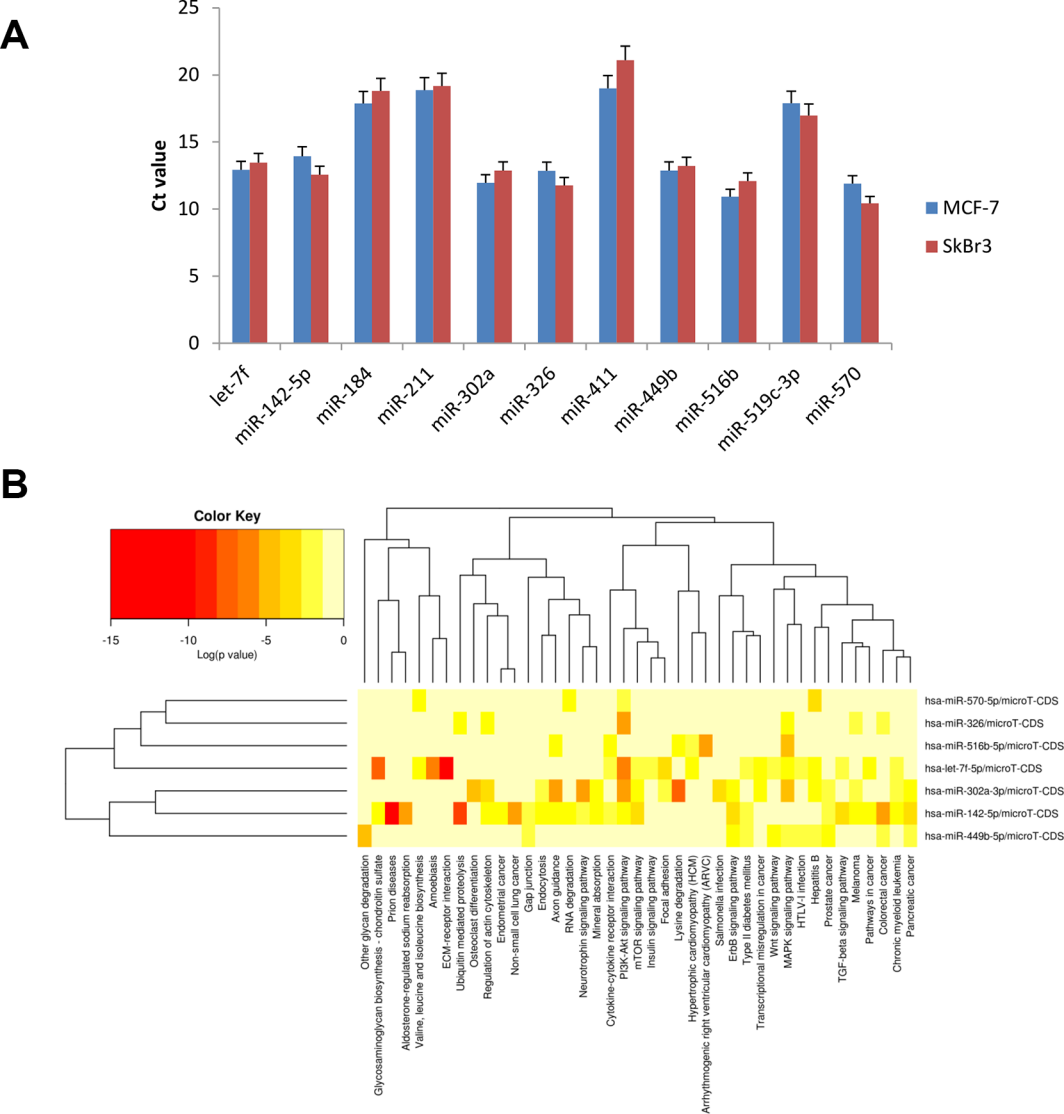
ZOL induces the expression of 11 specific miRNAs (**A**) Validation of miRNA array data by quantitative real-time PCR analysis. The mean Ct values of let-7f, miR-142-5p, miR-184, miR-211, miR-302a-3p, miR-326, miR-411, miR-449b-5p, miR-516b-5p, miR-519c-3p and miR-570-5p were determined in MCF-7 and SkBr3 cells treated with 10 μM ZOL for 24 h. RNU48 was used as endogenous control. Data are presented as Ct values ± SDs. Untreated cells showed undetermined values of Ct for all 11 miRNAs; (**B**) miRNAs versus pathways heat map (clustering based on significance levels). Darker colors represent lower significance values. The dendrograms placed on both axes depict hierarchical clustering results for miRNAs and pathways, respectively. On the miRNA axis, we can identify clustered miRNAs by exhibiting similar pathway targeting patterns. An analogous clustering can be observed also on the pathway axis. Hierarchical clustering was realized using DIANA-miRPath v2.0.

A further series of studies was conducted on the miRNAs expression profile data in order to identify any pathway of particular interest. Array data showed that ZOL treatment induces expression of 11 miRNAs in MCF7 cells. In order to validate miRNA expression, quantitative Real-Time PCR was performed on another pair of independent samples from MCF7 and SkBr3 cells using TaqMan miRNA assays. Data confirmed that 11 miRNAs were specifically expressed in both MCF7 and SkBr3 cells treated with 10 μM ZOL for 24 h compared to untreated cells (Figure 2A). Seven miRNAs were mainly expressed: let-7f, miR-142-5p, miR-302a-3p, miR-326, miR-449b-5p, miR-516b-5p and miR-570-5p (Table S1). Untreated cells showed undetermined values of Ct for all 11 miRNAs. To understand the biological role of these miRNAs we created heat maps of miRNAs versus pathways using miRPath v2.0.

The integrated analysis was performed on 7 miRNAs induced after ZOL treatment. This analysis indicated that miRNAs showing high expression levels were included in the following three pathways: PI3K/Akt signaling (hsa04151), MAPK signaling (hsa 04010) and regulation of actin cytoskeleton (hsa 04010) (Figure 2B). In particular, we found 5 miRNAs, among the 7 most representative miRNAs, implicated in the regulation of PI3K/Akt signaling pathway, suppressing the expression of 87 hypothetical genes; 5 miRNAs inhibiting 55 potential targets were involved in MAPK signaling cascade; and the pathway of actin cytoskeleton regulation was shared by 3 miRNAs modulating 40 potential candidate genes. In addition, the ubiquitin-mediated proteolysis (25 genes, 2 miRNAs), mTOR (16 genes, 3 miRNAs), Erb (20 genes, 3 miRNAs), and TGF-β (16 genes, 2 miRNAs) signaling pathways and focal adhesion molecules (25 genes, 2 miRNAs) were statistically relevant (Table 3).

**Table 3:** Cellular pathways modulated by specific miRNAs induced by ZOL in MCF7 and SkBr3 cells

Pathway	miRNAs	Genes
PI3K /Akt signaling pathway	5	87
MAPK signaling pathway	5	55
Regulation of actin cytoskeleton	3	40
Ubiquitin mediated proteolysis	2	25
mTOR signaling pathway	3	16
Erb signaling pathway	3	20
Focal adhesion	2	25
TGF-β signaling pathway	2	16

The pathways were obtained using DIANA-miRPath v2.0. The first column reports the pathway, the middle column shows the number of miRNAs involved in the same pathway and the last column reports the number of the target genes. *p* < 0.005.

### ZOL silences the expression of specific miRNAs

Our results showed that miRNA expression pattern of MCF-7 BC cells was distinctly different between untreated and treated cells with 10 μM ZOL for 24 h.

**Figure 3:**
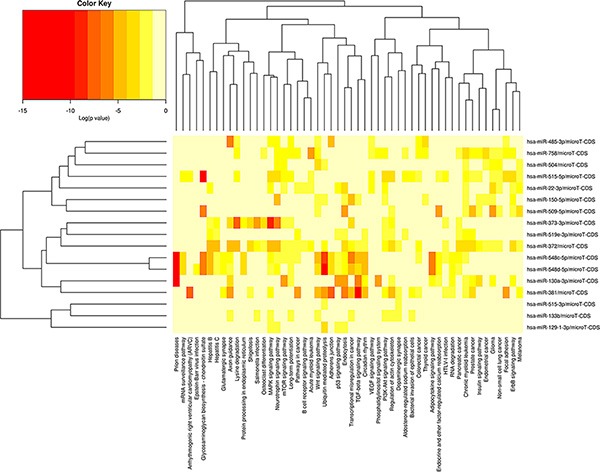
ZOL silences the expression of 22 miRNAs in breast cancer cells MiRNAs versus pathways heat map (clustering based on significance levels). Darker colors represent lower significance values. The dendrograms placed on both axes depict hierarchical clustering results for miRNAs and pathways, respectively. On the miRNA axis, we can identify clustered miRNAs by exhibiting similar pathway targeting patterns. An analogous clustering can be observed also on the pathway axis. Hierarchical clustering was realized using DIANA-miRPath v2.0.

Microarray data indicated that 22 miRNAs were specifically detected in untreated MCF-7 cells, but not in cells exposed to 10 μM ZOL for 24 h (Table S2). These results were confirmed in independent samples from both MCF7 and SkBr3 cells by means of quantitative Real-Time PCR analyses (data not shown). In order to elucidate the biological role of these silenced miRNAs, we created a miRNAs versus pathway heat map (Figure 3).

The integrated analysis was performed on miR-129-3p, miR-130a, miR-133b, miR-150, miR-22, miR-372, miR-373, miR-381, miR-485, miR-504, miR-509-5p, miR-515-3p, miR-515-5p, miR-519e, miR-548c-5p, miR-548d-3p and miR-758. In this analysis we have not considered miR-124, miR-187, miR-337-5p, miR-487a and miR-518d-5p because they have not been shown to share potential gene targets. The results showed that 10 miRNAs could modulate 95 gene targets involved in MAPK signaling cascade and 9 miRNAs could regulate genes involved in PI3K/Akt signaling pathway (Table 4). The p38-mitogen-activated protein kinase (MAPK) pathway is involved in the mechanism of the antitumor effect of ZOL according to our previous results [26]. ZOL mediated growth inhibition of BC cells in a dose- and time-dependent manner [26], regulated by changes in expression and/or membrane localization of Ras, Rap1, and phosphorylated MAPK [52]. Moreover, we found that 10 miRNAs, modulating 72 potential gene targets, were implicated in the regulation of endocytosis, and 35 genes involved in TGF-β signaling pathway could be modulated by 6 miRNAs. In addition, statistically relevant pathways were: Wnt signaling (58 genes, 9 miRNAs), ubiquitin-mediated proteolysis (49 genes, 8 miRNAs) and regulation of actin cytoskeleton (74 genes, 8 miRNAs) (Table 4).

**Table 4:** Cellular pathways modulated by silenced miRNAs in MCF7 and SkBr3 cells treated with ZOL

Pathway	miRNAs	Genes
MAPK signaling pathway	10	95
PI3K-Akt signaling pathway	9	106
Endocytosis	10	72
TGF-β signaling pathway	6	35
Wnt signaling pathway	9	58
Ubiquitin mediated proteolysis	8	49
Regulation of actin cytoskeleton	8	74

The pathways were obtained using DIANA-miRPath v2.0. The first column reports the pathway, the middle column shows the number of miRNAs involved in the same pathway and the last column reports the number of the target genes. *p* < 0.005.

## DISCUSSION

Nowadays, although clinicians have several treatment options (chemotherapy, hormone therapy, and targeted therapy), BC is still responsible for a significant percentage of cancer deaths in women [53, 54]. ZOL showed anti-tumoral and anti-metastatic activity during cancer progression in preclinical and clinical studies [55]. Several clinical trials analyzed the effects of ZOL on overall survival of BC patients [56, 57]. The mechanism by which ZOL explicates its antitumor properties has already been studied, and its inhibitory effect on tumor angiogenesis has been demonstrated. It has been reported that proliferation, migration and invasion are inhibited by ZOL in order to promote apoptosis and reduce the adhesion to bone of malignant cells [26, 58, 59].

The main aim of our study was to investigate the molecular mechanisms by which ZOL exerts its anti-tumoral effects in BC cells focusing our attention on miRNAs. Based on the TaqMan Low density array analysis, we identified 54 differentially expressed miRNAs in human MCF-7 BC cells after treatment with 10 μM ZOL for 24 h with different abundance for each miRNA. Most of these miRNAs has not been reported or investigated in BC cells. Our analyses showed that low-dose ZOL treatment affected the expression levels of some miRNAs in MCF-7 cells. In order to identify the cellular pathways modulated by deregulated miRNAs we performed an integrated analysis using mirPath software. MiRNAs versus pathway heat map showed that, among the 21 deregulated miRNAs, 6 miRNAs shared 60 genes involved in the PI3K-Akt signaling pathway. Lysine degradation, Wnt signaling, TGF-β signaling, Jak-STAT signaling, regulation of actin cytoskeleton and mTOR signaling were other significantly relevant pathways identified by statistical analysis. We also found that 10 μM ZOL treatment for 24h induced expression of 11 specific miRNAs in MCF7 and SkBr3 cells. We focused our attention on 7 miRNAs which were more expressed than others: let-7f, miR-142-5p, miR-302a-3p, miR-326, miR-449b-5p, miR-516b-5p and miR-570-5p.

The integrated analysis indicated that miRNAs showing high expression levels were involved in PI3K/Akt signaling, MAPK cascade, regulation of actin cytoskeleton, ubiquitin-mediated proteolysis, mTOR, TGF-β and Erb signaling pathways, and focal adhesion molecules.

In contrast, 22 miRNAs were expressed in untreated MCF7 and SkBr3 cells only, indicating that low-dose ZOL treatment silences these miRNAs, probably regulating transcriptional activation factors. Integrated analysis showed that 17 miRNAs could modulate genes involved in MAPK, PI3K/Akt, TGF-β and Wnt signaling pathways, ubiquitin-mediated proteolysis, and regulation of actin cytoskeleton. Overall, miRNAs regulated by low doses of ZOL could modulate genes involved in cancer-related pathways.

Additionally, a comparison between the microRNA expression profile described in our work and that obtained in other recent papers concerning the involvement of miRNAs in bone metastasis formation showed that low-dose ZOL treatment up-regulates three miRNAs opposing to metastasis development (miR-143, miR-145 and miR-204), induces specific expression of miR-211 (suppressor of osteoclast function), and specifically suppresses the expression of miR-373 (metastasis promoter). Furthermore, our data showed a ZOL-induced down-regulation of the miR-96 expression, whose up-regulation, conversely, has been shown to increase cell proliferation in human BC via direct targeting of *FOXO3a* [60]. In addition, our analyses revealed a specific ZOL-mediated induction of expression of miR-302a and miR-326, that is perfectly coherent with the recent findings from Liang and collaborators demonstrating the involvement of these miRNAs in invasion and metastasis, and therapy resistance, respectively, in BC [61, 62]. Indeed, the restoration of the miR-302a expression has been shown to inhibit the invasive ability and metastasis both *in vitro* and *in vivo* by down-regulation of CXCR4 expression [61], whereas the ectopic expression of miR-326 sensitized multidrug-resistant BC cells to chemotherapy by down-regulation of MRP-1 expression [62]. Lastly, since Pandey et al. [63] recently reported that high expression levels of miR-22 are associated with progression, metastasis and poor prognosis in BC patients, our results showing the ZOL-mediated silencing of miR-22 expression support the already known role of ZOL as effective anticancer agent.

Designing therapies targeting the metastasis development mechanisms could prevent the escape of BC cells from a primary tumor and inhibit bone metastasis formation. The current study demonstrates that aberration of specific miRNAs after ZOL treatment may be effector of the anti-tumoral activity of ZOL in BC cells.

The microRNA expression profile obtained in this work is correlated with cancer-related biological pathways, such as PI3K/Akt, MAPK, TGF-β signaling and actin cytoskeletal remodeling. This data is in agreement with that reported in our previous work [26], where we confirmed, using the same ZOL concentration, the phosphorylation inhibition of the AKT and MAPK proteins. The obtained results are indicative of the mechanisms by which ZOL is able to inhibit cellular proliferation [26]. Also, we found that ZOL plays an inhibitory role in BC cell invasion through cytoskeletal remodeling. The molecular mechanism underlying this effect is the activation of TGF-β1/Smad signaling pathway and downstream activity of FN1 and β-actin. In future, investigating the molecular regulatory mechanism of drug-specific miRNAs will allow a better understanding of the action mechanism of ZOL and the detection of novel potential targets useful for the development of possible new therapeutic strategies.

## MATERIALS AND METHODS

### Cell culture

Human BC cell lines, MCF-7 and SkBr3, purchased from the American Type Culture Collection (Rockville, MD, USA) were grown in Dulbecco's modified Eagle's medium Gibco DMEM:F12 (Invitrogen, Carlsbad, CA, USA) containing 10% fetal bovine serum (FBS) and 1% Penicillin/Streptomycin (P/S) (Gibco). Cells were incubated at 37°C in a humidified atmosphere of 5% of CO_2_. Eighty per cent confluent cultures were stimulated with ZOL 10 μM for 24 h. ZOL was kindly provided by Novartis Pharma AG.

### MiRNA expression profile analysis

Total cellular RNA and miRNAs has been isolated using the miRNeasy Mini Kit (Qiagen Inc, Valencia, CA). The quality of the samples have been controlled through RNA 6000 Nano Assay (Agilent Techologies, Palo Alto, CA, USA) using 2100 Bioanalyzer (Agilent Technologies, Santa Clara, CA) and quantified through the spectrophotometer NanoDrop ND-1000 (CELBIO). To study miRNA expression profile, we used TaqMan^®^ Low Density Array A Human MicroRNA v2.0 (Life Technologies, Carlsbad, California, U.S.). The arrays were performed in accordance to manufacturer's protocols as previously described [64]. Briefly, 600 ng of miRNA-enriched total RNA were reverse transcribed using Megaplex^™^ RT Primers Human Pool A (Life Technologies, Carlsbad, California, U.S.) according to manufacturer's instructions. Conditions for the reverse transcription reaction were as follows: 16°C for 2 minutes, 42°C for 1 minute, 50°C for 1 second for 40 cycles, 85°C for 5 minutes then hold at 4°C. Obtained cDNA was diluted, mixed with TaqMan Gene Expression Master Mix, and loaded into each of the eight fill ports on the TaqMan^®^ Human MicroRNA Array A (Life Technologies, Carlsbad, California, U.S.). The TaqMan Human MicroRNA Array is a 384-well microfluidics card containing 377 primer-probe sets for individual miRNAs as well as three carefully selected candidate endogenous small nucleolar RNAs control assay and one negative control assay. The array was centrifuged at 1,200 rpm twice for 1 minute each, then run on ABI-PRISM 7900 HT Sequence Detection System (Applied Biosystems). Two biological replicates were performed for each experimental condition. The data were quantified using the SDS 2.4 software and normalized using the RNU48 as endogenous control. The cycle threshold (Ct) value, which was calculated relatively to the endogenous control, was used for our analysis (ΔCt). The 2^−ΔΔCT^ (delta-delta-Ct algorithm) method was used to calculate the relative changes in miRNA expression. A miRNA was defined differentially expressed when estimated *P*-value was < 0.05.

### Quantitative real-time PCR

Ten nanograms of total RNA from another independent experiment were reverse transcribed using Taqman MicroRNA Reverse Transcription Kit (Life Technologies, Carlsbad, California, U.S.) according to manufacturer's instructions. The obtained cDNA was amplified using the following Taqman MicroRNA assays: hsa-let-7f-5p, hsa-miR-142-5p, hsa-miR-184, hsa-miR-211, hsa-miR-302a, hsa-miR-326, hsa-miR-411, hsa-miR-449b, hsa-miR-516b, hsa-miR-519c-3p and hsa-miR-570 (Life Technologies, Carlsbad, California, U.S.). To normalize quantitative Real-Time PCR reactions, parallel reactions were run on each sample for RNU48 snRNA. The reactions were performed in triplicate and changes in the target miRNA content relative to RNU6B were determined using the comparative Ct method to calculate changes in Ct, and, ultimately, fold and percent change. An average Ct value for each RNA was obtained for replicate reactions.

### MiRNA data analysis

Hierarchical cluster and heat map analyses were performed using the MultiExperiment Viewer (MeV v4.8) program of TM4 Microarray Software Suite. Heat maps of miRNAs versus pathways were generated using miRPath v2.0 database as previously described [43, 65]. DIANA-miRPath v2.0 is based on a new relational schema, specifically designed to accommodate this as well as future miRPath updates. MiRNA and pathway related information was obtained from miRBase 18 [66] and Kyoto Encyclopedia of Genes and Genomes (KEGG) v58.1 [67]. Hierarchical clustering of targeted pathways and miRNAs was realized using DIANA-miRPath v2.0. The software created a clustering of the selected miRNAs based on their influence on molecular pathways [65].

### Statistical analysis

Filtering criteria able to select reliably quantifiable miRNAs were used (cut off < 35 Ct). Undetermined values of Ct were estimated as 40 Ct (the last cycle of the reactions). Heat maps were constructed using z-transformed relative gene ΔCt values, so that measurements were scaled to obtain gene-wise zero mean and unit variance. Data are represented as mean ± S.D (standard deviation). Statistical analyses were performed by Student's *t*-test. Values of *P* < 0.05 were considered to be statistically significant.

## SUPPLEMENTARY MATERIALS FIGURES AND TABLES


