# Supplementary material for: Can the microRNA expression profile help to identify novel targets for zoledronic acid in breast cancer?
Source: Oncotarget. 2016 Apr 13;7(20):29321–32. doi: 10.18632/oncotarget.8722 (PMC5045398; doi:10.18632/oncotarget.8722)
Supplement: Supplementary file 1 [file oncotarget-07-29321-s001.pdf]

Can the microRNA expression profile help to identify novel targets for zoledronic acid in breast cancer?

Supplementary Materials

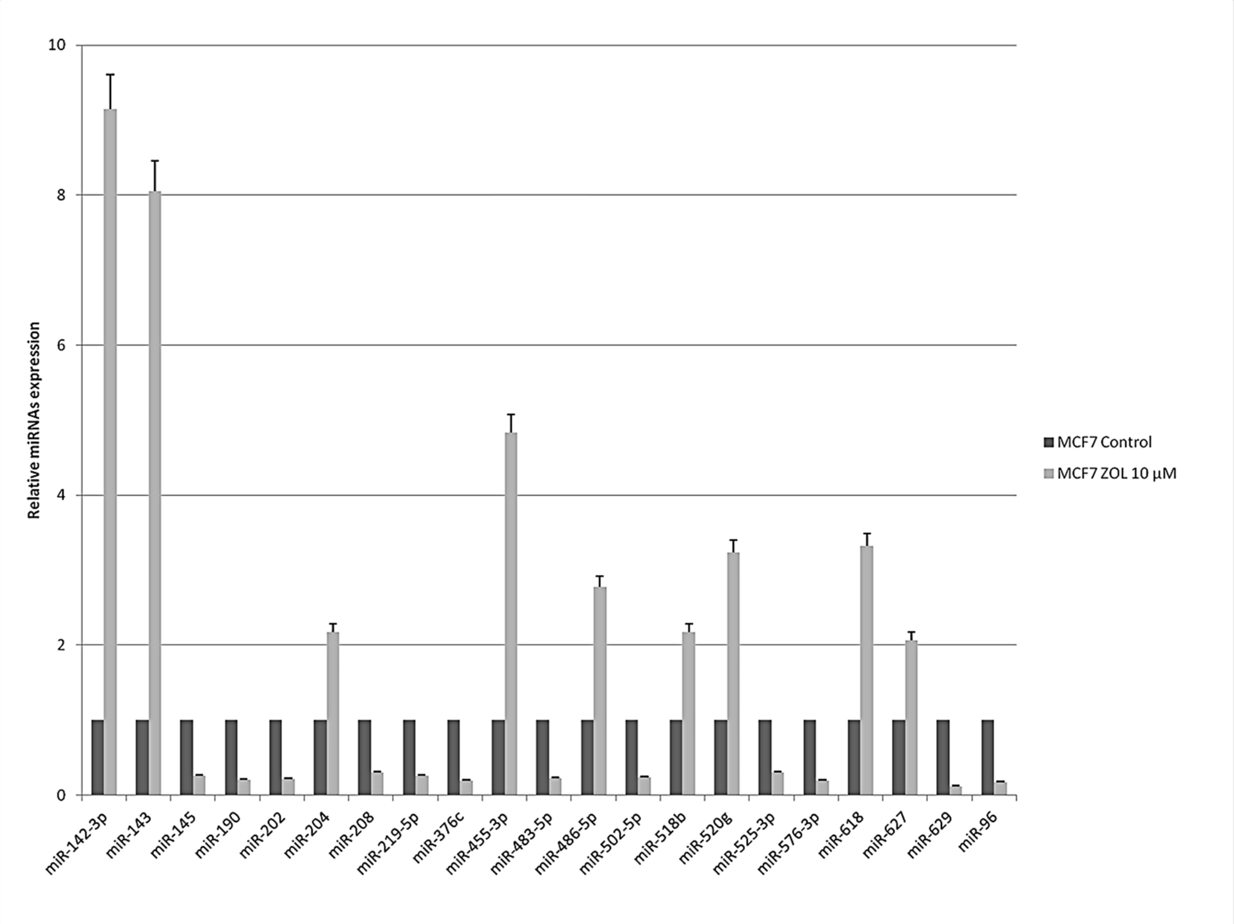

Supplementary Figure S1: Low-dose ZOL up-regulates 9 miRNAs and down-regulates 12 miRNAs. Relative expression of miRNAs deregulated by ZOL. The graph reports the relative mean value of 9 up-regulated and 12 down-regulated miRNAs in MCF7 cells treated with 10 µM ZOL for 24 h compared to untreated cells (MCF7 control). Data was obtained by TaqMan® Low Density Array A Human MicroRNA using RNU48 as endogenous control.

Supplementary Table S1: List of the 11 miRNAs induced by low-dose ZOL treatment

miRNAs	MCF7 Ct value	SkBr3 Ct value
let-7f	12.92	13.47
miR-142-5p	13.95	12.56
miR-184	17.88	18.8
miR-211	18.86	19.17
miR-302a	11.96	12.87
miR-326	12.85	11.76
miR-411	19.00	21.09
miR-449b	12.87	13.21
miR-516b	10.93	12.09
miR-519c-3p	17.90	16.98
miR-570	11.89	10.43

Supplementary Table S2: miRNAs expressed in control samples only

miR-124	miR-485
miR-129-3p	miR-487a
miR-130a	miR-504
miR-133b	miR-509-5p
miR-150	miR-515-3p
miR-187	miR-515-5p
miR-22	miR-518d-5p
miR-337-5p	miR-519e
miR-372	miR-548c-5p
miR-373	miR-548d-3p
miR-381	miR-758
